# Supplementary material for: Overcome low levels of detection limit and choice of antibody affects detection of lipoarabinomannan in pediatric tuberculosis
Source: PLoS One. 2022 Oct 11;17(10):e0275838. doi: 10.1371/journal.pone.0275838 (PMC9553055; doi:10.1371/journal.pone.0275838)
Supplement: S1 Data — (DOCX) [file pone.0275838.s001.docx]

**Overcome Low Levels of Detection Limit and**

**Choice of Antibody Affects Detection of Lipoarabinomannan in Pediatric Tuberculosis**

Anita G. Amin^1a^, Prithwiraj De^1a^, Barbara Graham^1^, Brooke L. Jensen^1^, Emmanuel Moreau^2^ , and

Delphi Chatterjee^1*^

^1^Mycobacteria Research Laboratory, Department of Microbiology, Immunology, and Pathology, Colorado State University, Fort Collins, CO 80523, USA.

^2^ Foundation of Innovative New Diagnostics, Geneva, Switzerland

**^a^ Equal contributions**

* **Corresponding Author**

E.Mail: [delphi.chatterjee@colostate.edu](mailto:delphi.chatterjee@colostate.edu); Tel: +1 970 491 7495

**Coauthors email addresses**

[anita.amin@colostate.edu](mailto:anita.amin@colostate.edu)

[prithwiraj.de@colostate.edu](mailto:prithwiraj.de@colostate.edu)

[barb.graham@colostate.edu](mailto:barb.graham@colostate.edu)

[Brooke.Jensen@colostate.edu](mailto:Brooke.Jensen@colostate.edu)

[emmanuel.moreau@finddx.org](mailto:emmanuel.moreau@finddx.org)

emmanuel.moreau2008@yahoo.ca (present email)

Key words: Tuberculosis, Lipoarabinomannan, FujiLAM, Pediatric TB, Diagnostic

**Table of Contents**

|  | \| **PAGE** \| **FIGURE** \| **DESCRIPTION** \| \| --- \| --- \| --- \| \| S-0 \| Word doc. \| Table of Contents \| \| S-3 to S-19 \| Fig S1 -S13 \| GC/MS Chromatograms of TBSA Analysis of 91 pediatric urine samples includes both culture positive and culture negative samples. \| \| S-20 to S-22 \| Table SI  Table SII \| GC/MS Data and ELISA OD_450_ values, clinical status on all 91 pediatric urine samples  ELISA OD_450_ values and clinical status for 25 adult urine samples \| \| S-23 \| Fig S14 \| forest Plot- Age vs LAM distribution using BJ76 \| |  |
| --- | --- | --- | --- | --- | --- | --- | --- | --- | --- | --- | --- | --- | --- | --- | --- | --- | --- |
|  |  |  |

.
