# Supplementary material for: Overcome low levels of detection limit and choice of antibody affects detection of lipoarabinomannan in pediatric tuberculosis
Source: PLoS One. 2022 Oct 11;17(10):e0275838. doi: 10.1371/journal.pone.0275838 (PMC9553055; doi:10.1371/journal.pone.0275838)
Supplement: S1 File — GC/MS chromatograms of TBSA analysis of 91 pediatric urine samples includes both culture positive and culture negative samples. (PPTX) [file pone.0275838.s002.pptx]

## Slide 1
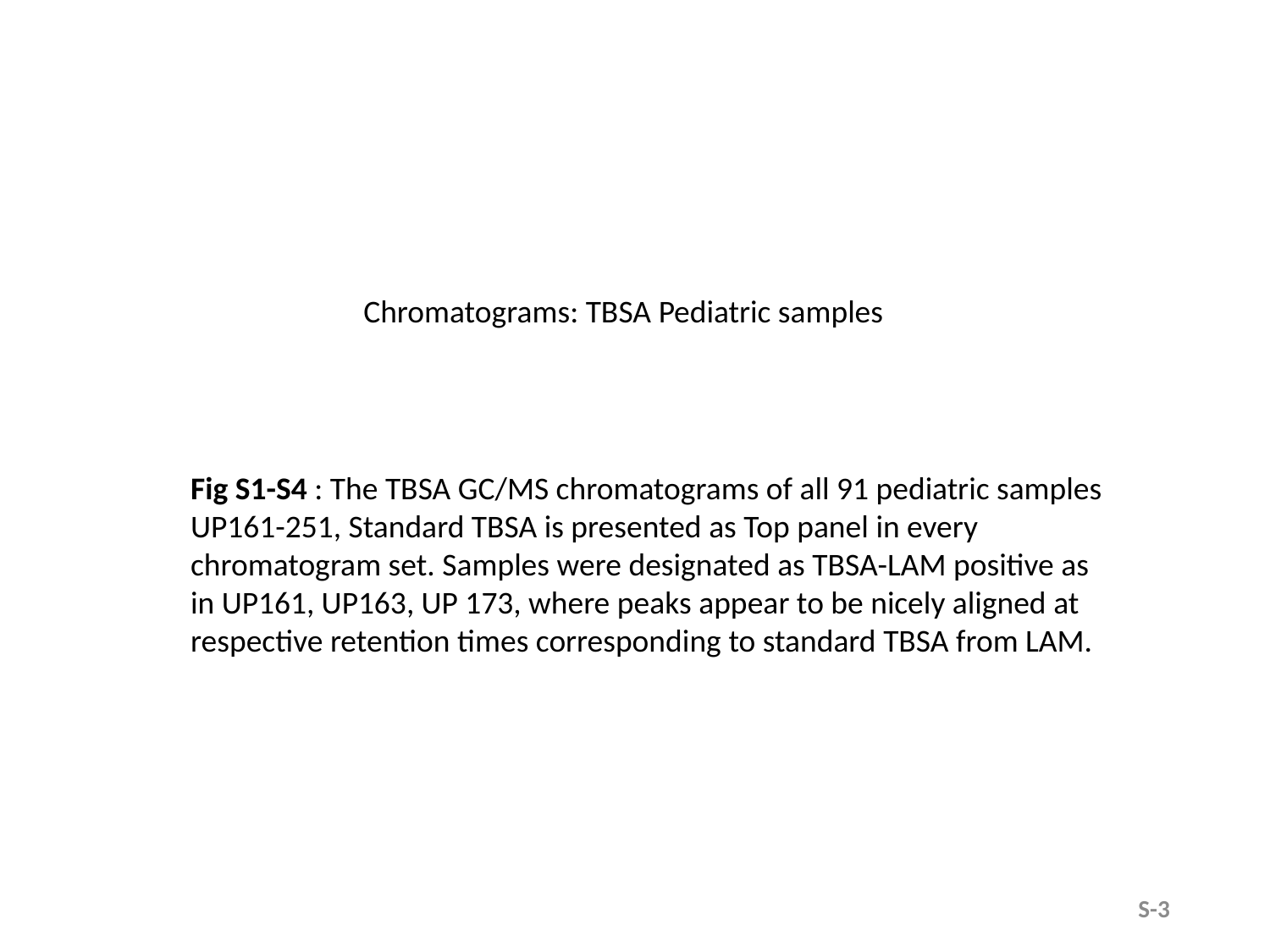

Chromatograms: TBSA Pediatric samples
Fig S1-S4 : The TBSA GC/MS chromatograms of all 91 pediatric samples UP161-251, Standard TBSA is presented as Top panel in every chromatogram set. Samples were designated as TBSA-LAM positive as in UP161, UP163, UP 173, where peaks appear to be nicely aligned at respective retention times corresponding to standard TBSA from LAM.
S-3

## Slide 2
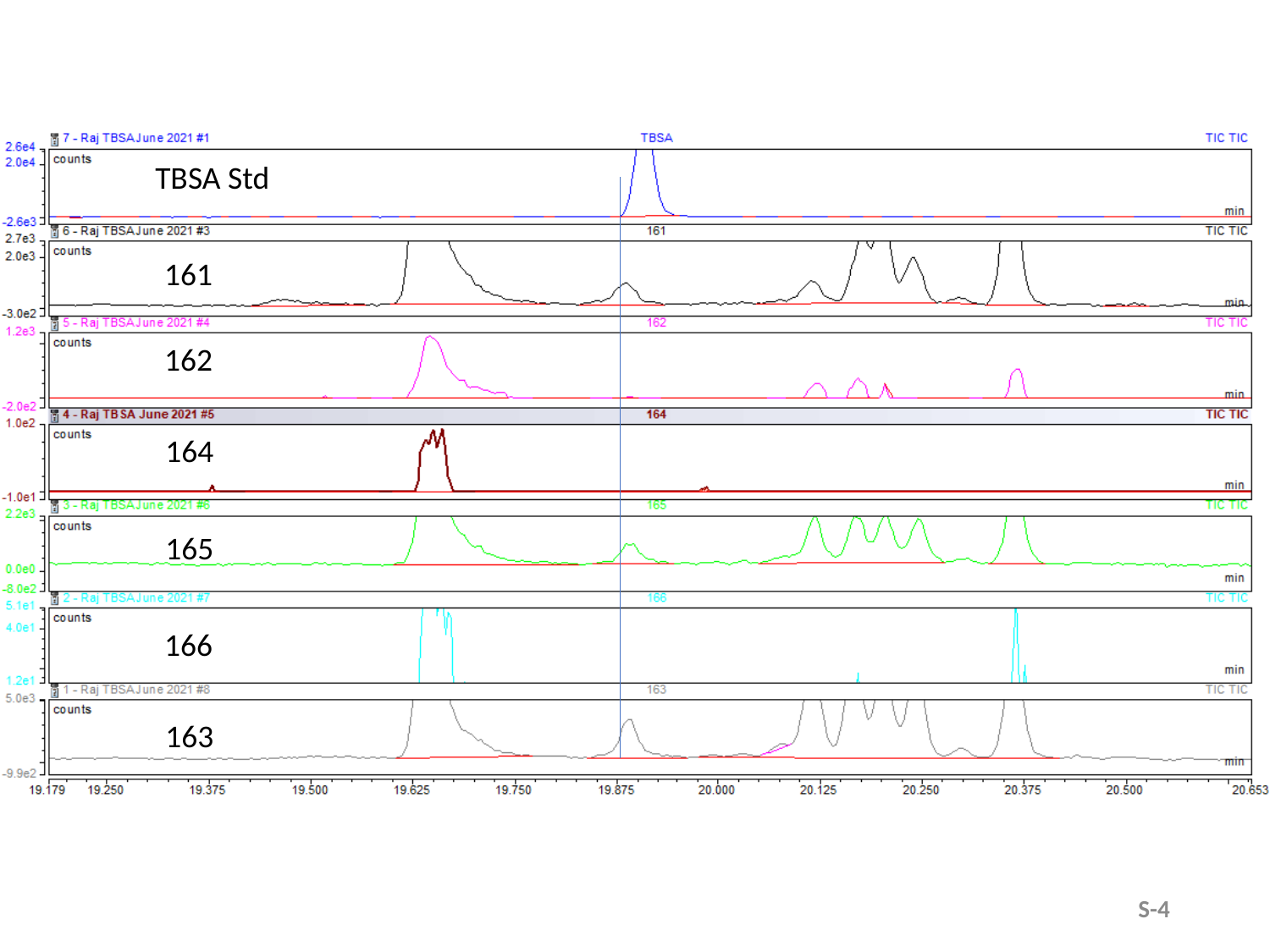

TBSA Std
161
162
164
165
166
163
S-4

## Slide 3
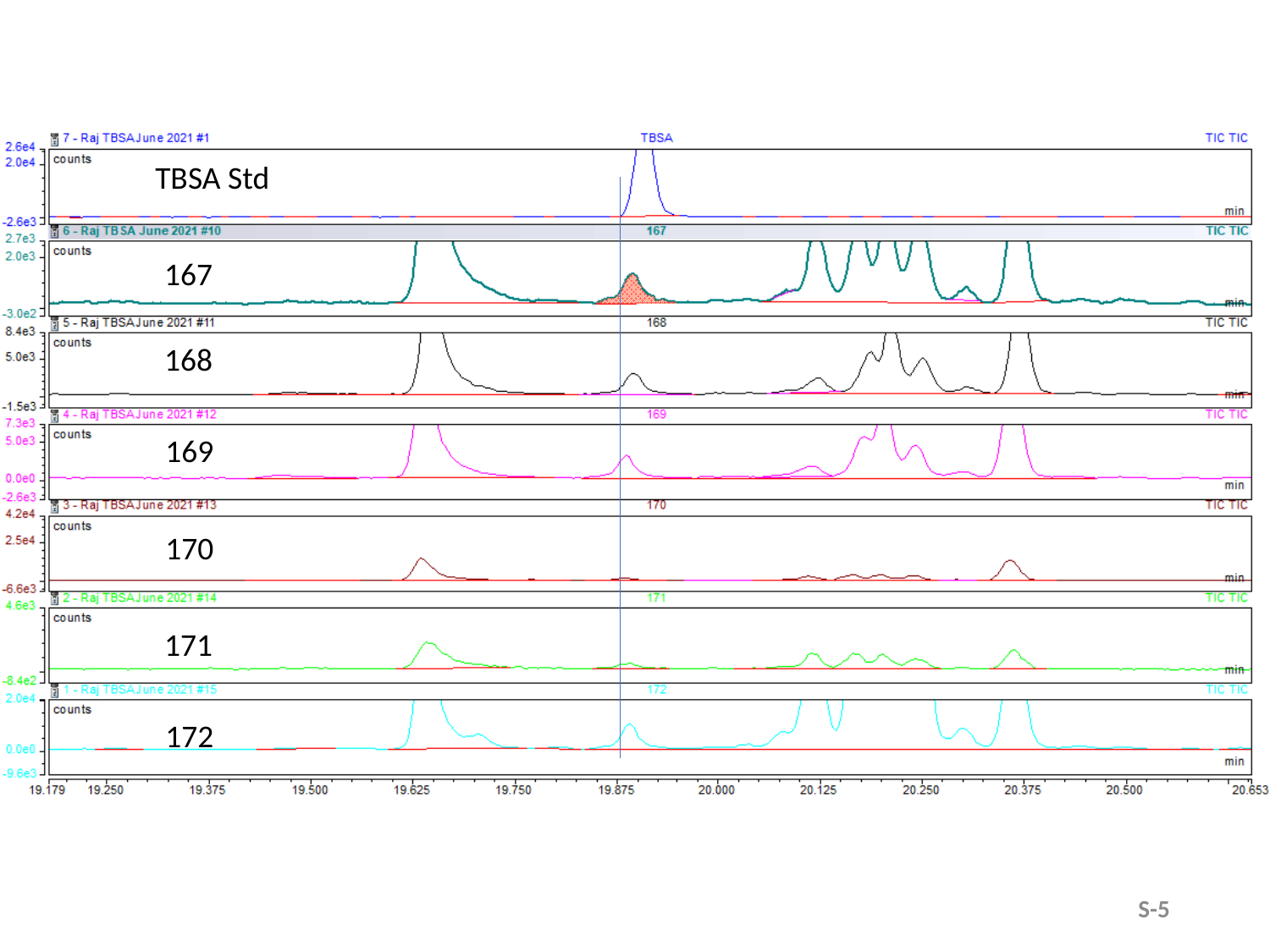

TBSA Std
167
168
169
170
171
172
S-5

## Slide 4
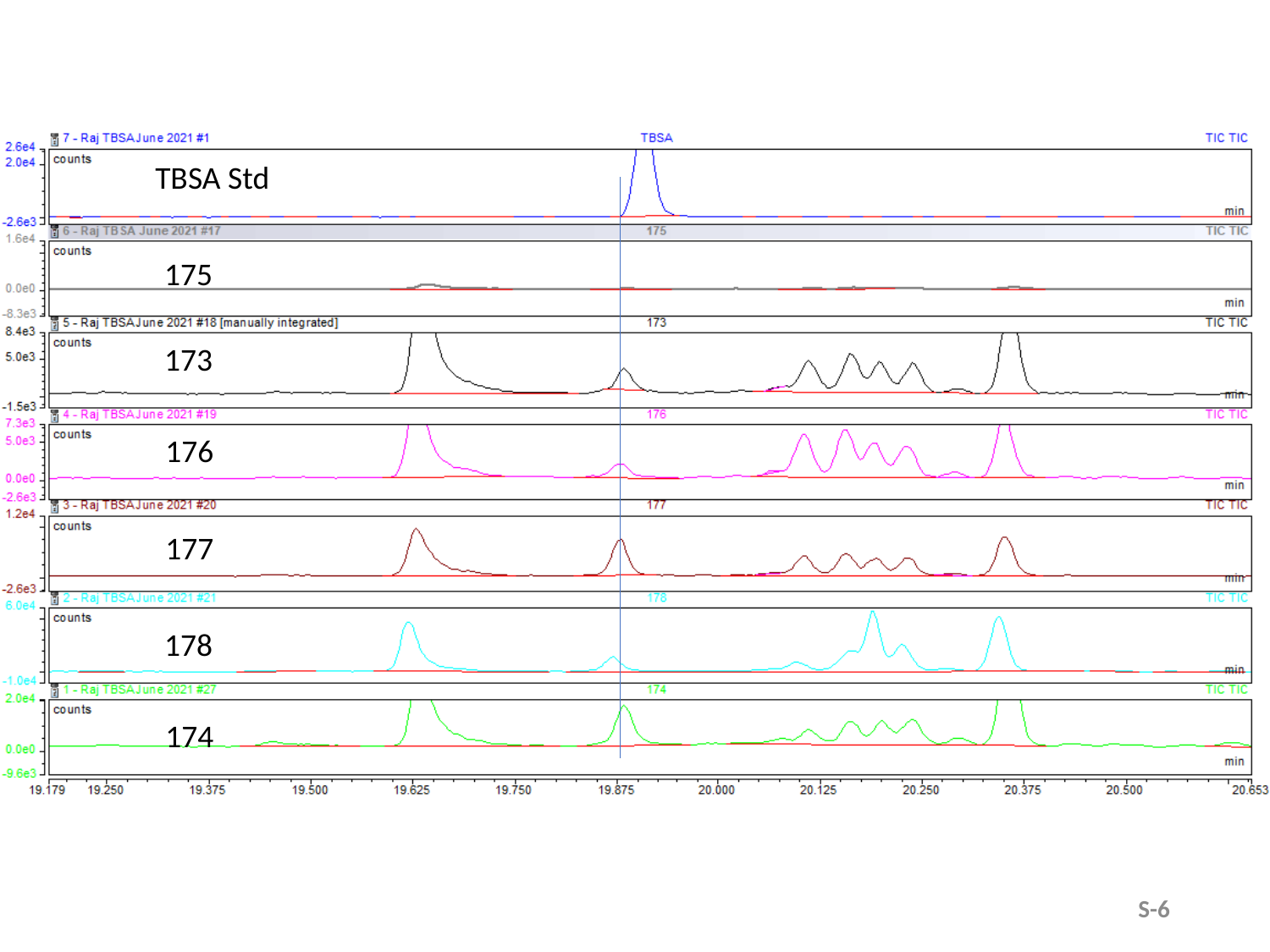

TBSA Std
175
173
176
177
178
174
S-6

## Slide 5
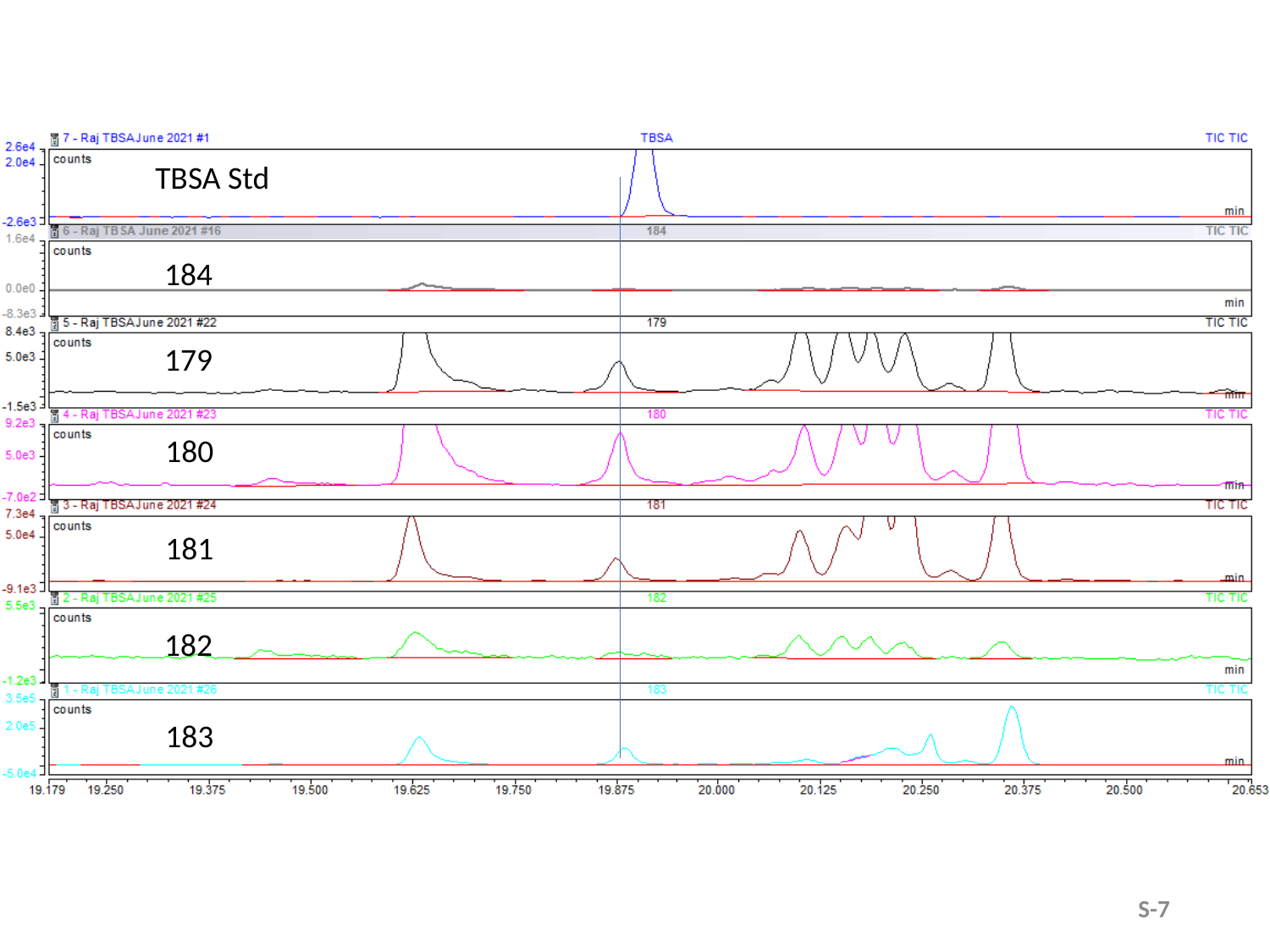

TBSA Std
184
179
180
181
182
183
S-7

## Slide 6
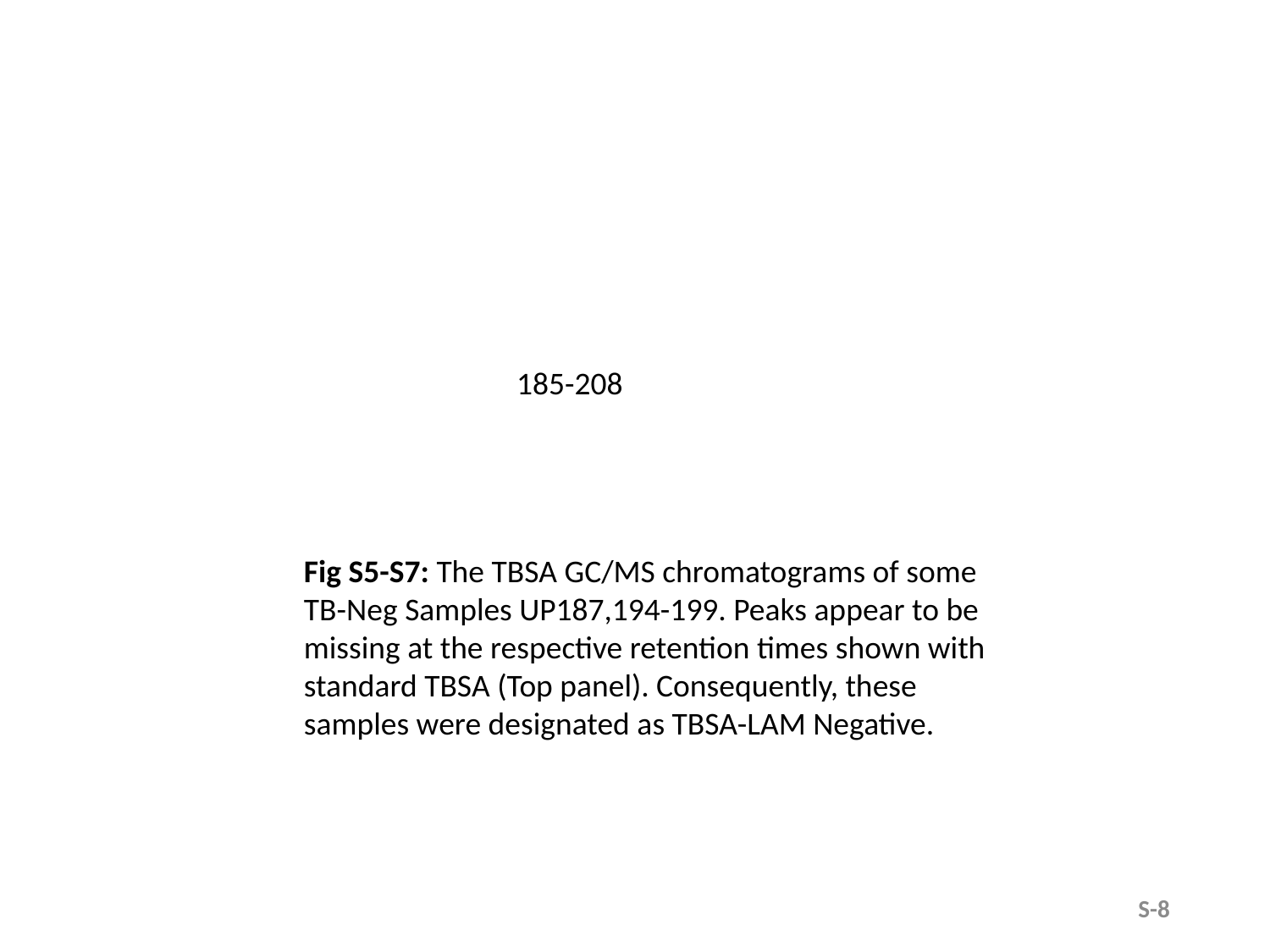

185-208
Fig S5-S7: The TBSA GC/MS chromatograms of some TB-Neg Samples UP187,194-199. Peaks appear to be missing at the respective retention times shown with standard TBSA (Top panel). Consequently, these samples were designated as TBSA-LAM Negative.
S-8

## Slide 7
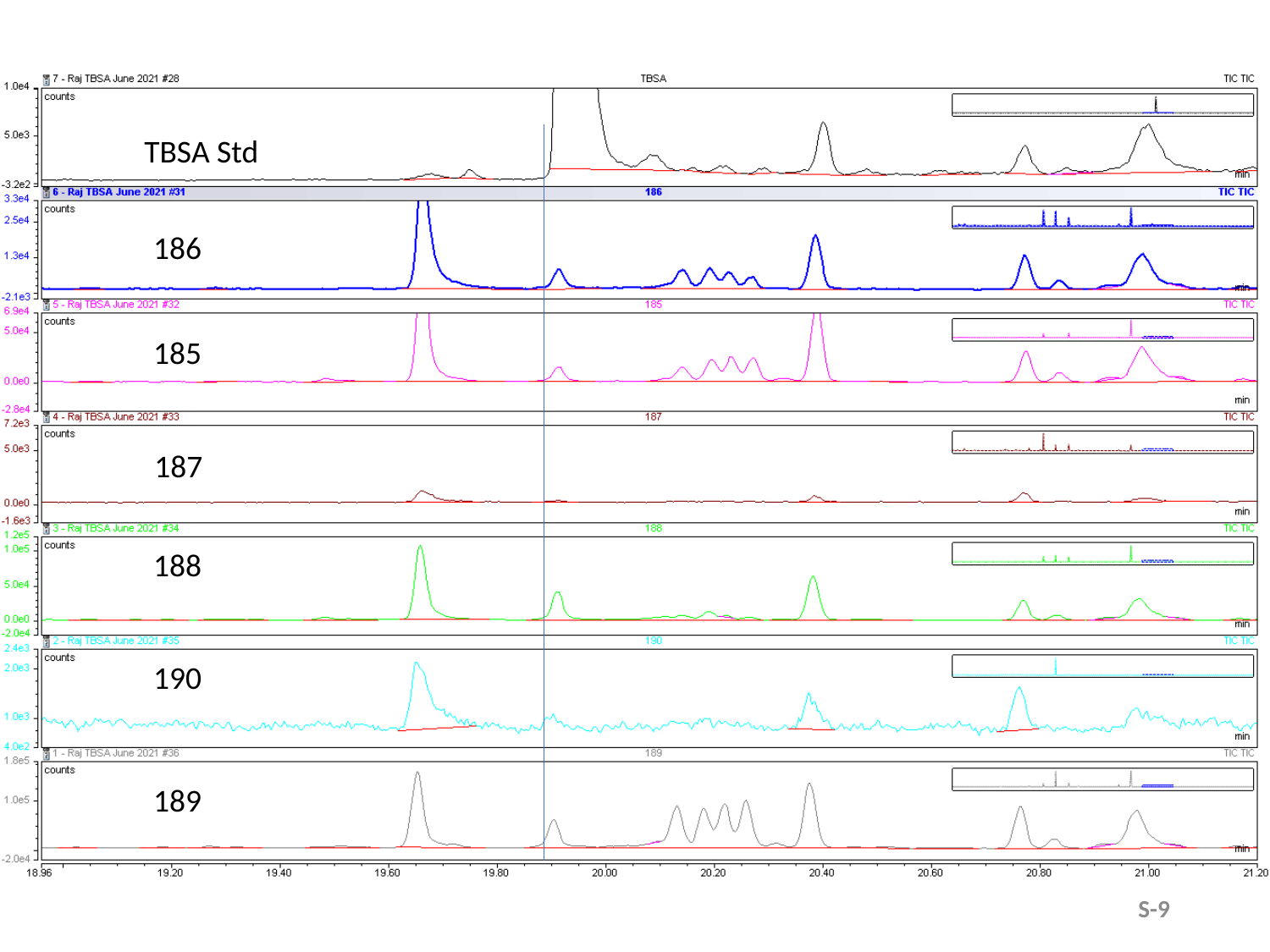

TBSA Std
186
185
187
188
190
189
S-9

## Slide 8
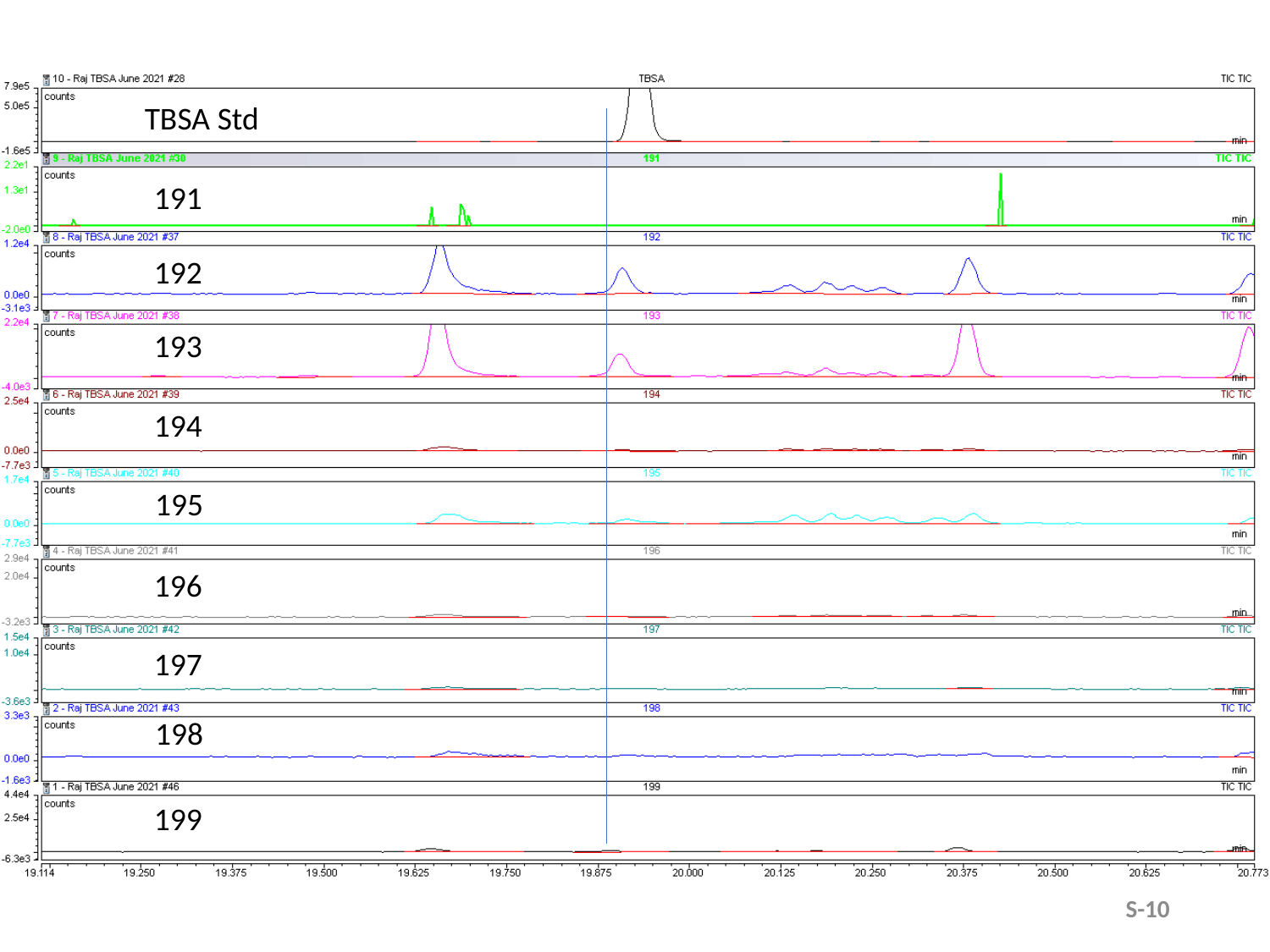

TBSA Std
191
192
193
194
195
196
197
198
199
S-10

## Slide 9
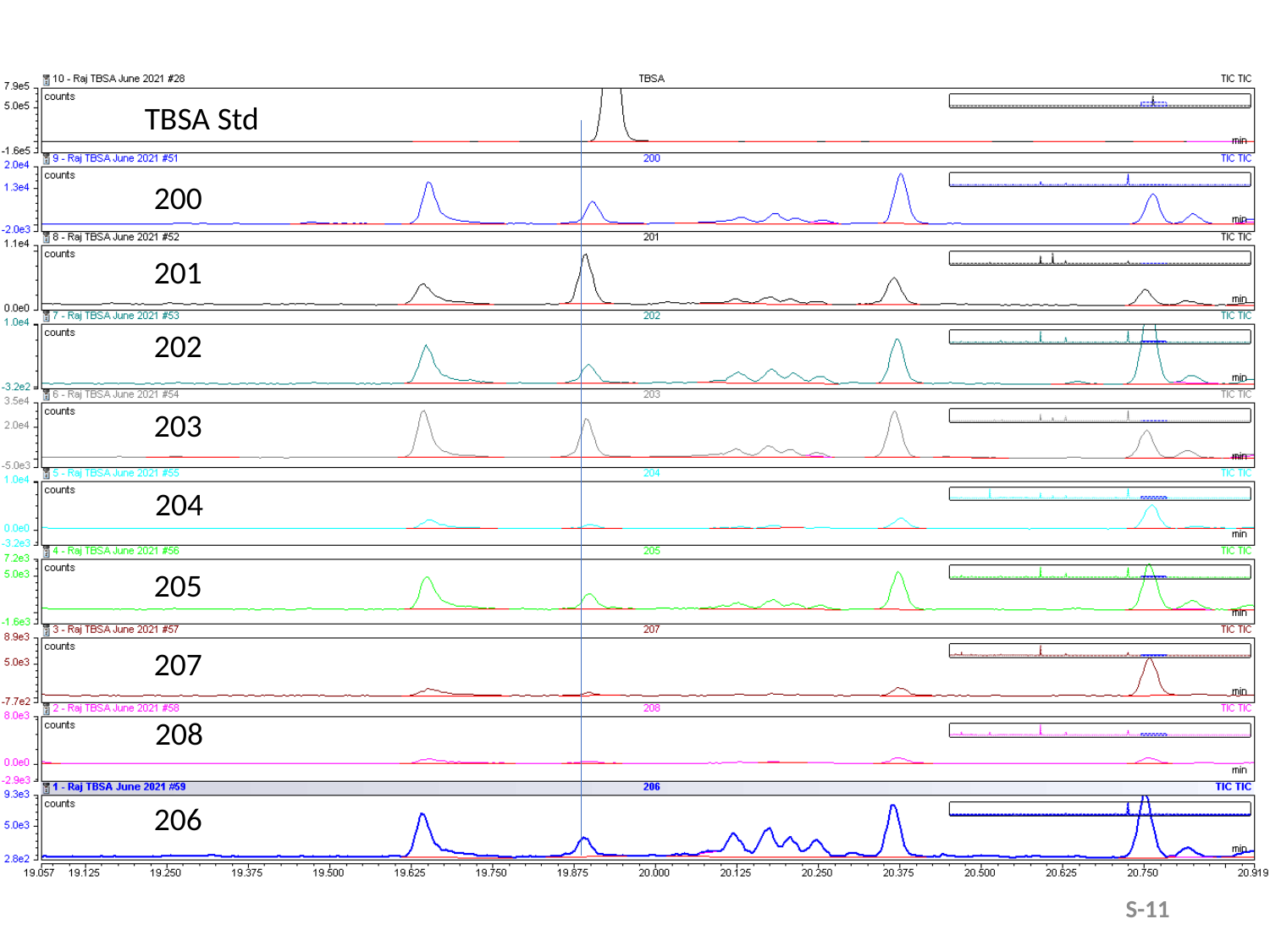

TBSA Std
200
201
202
203
204
205
207
208
206
S-11

## Slide 10
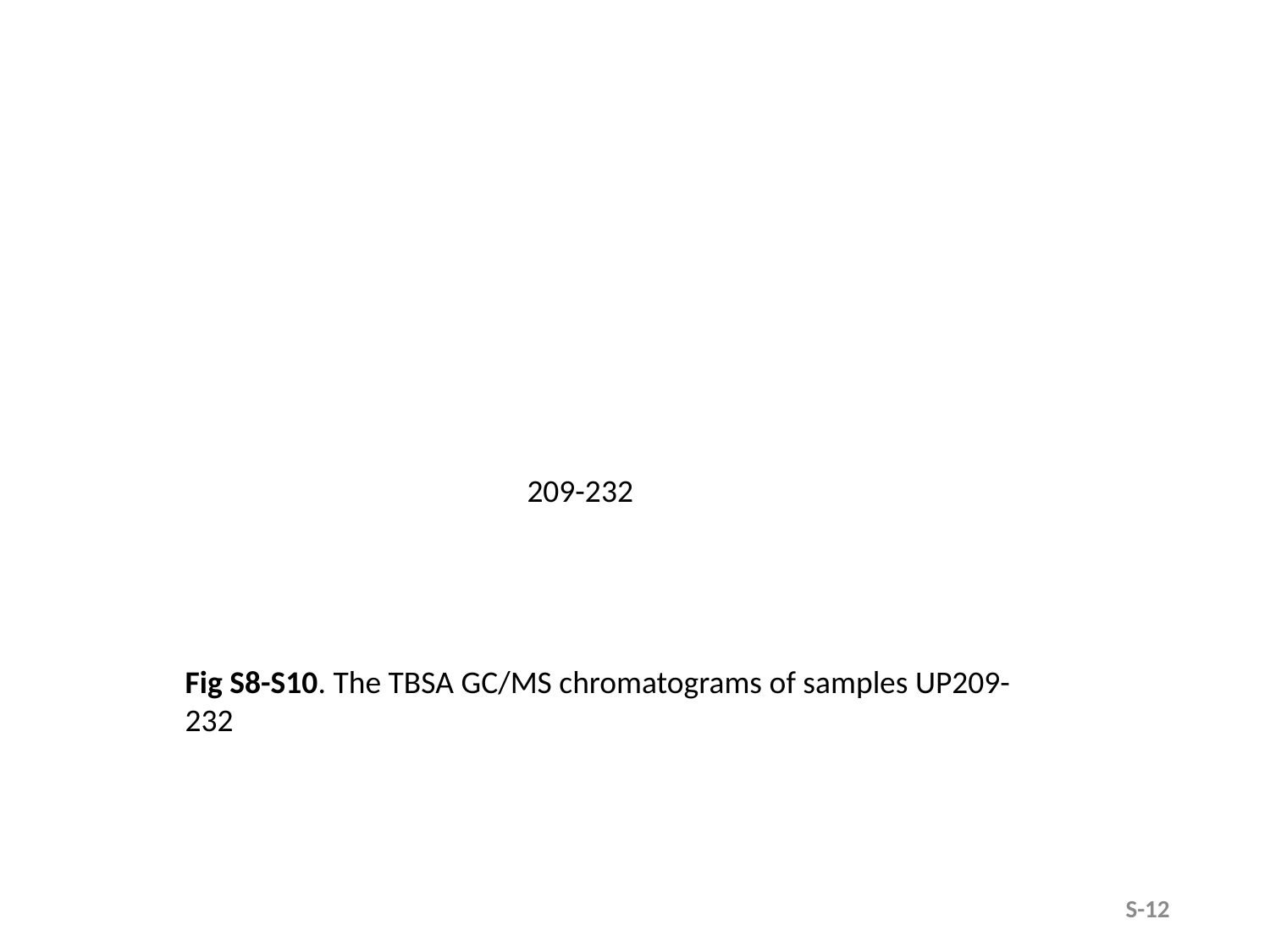

209-232
Fig S8-S10. The TBSA GC/MS chromatograms of samples UP209-232
S-12

## Slide 11
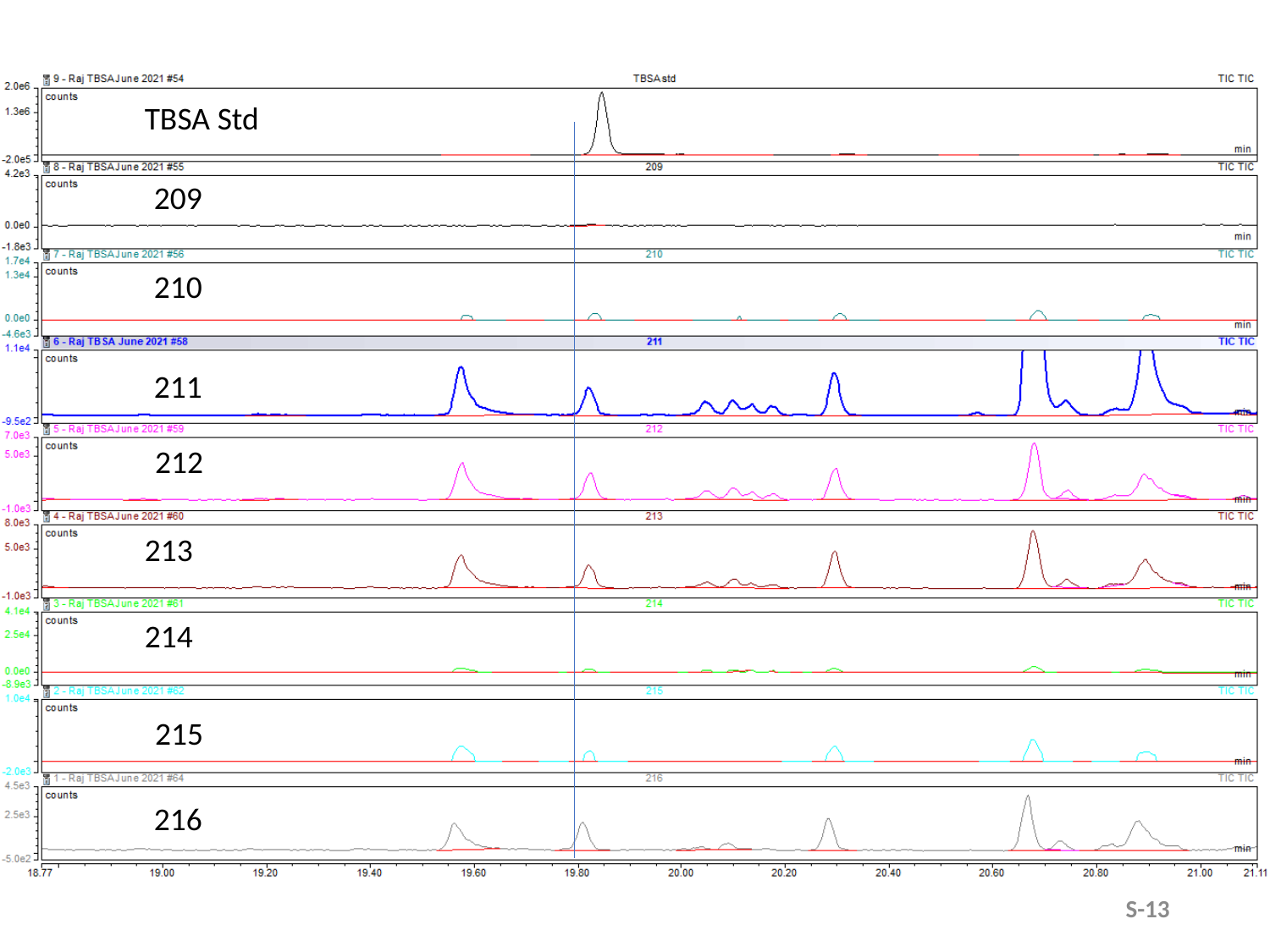

TBSA Std
209
210
211
212
213
214
215
216
S-13

## Slide 12
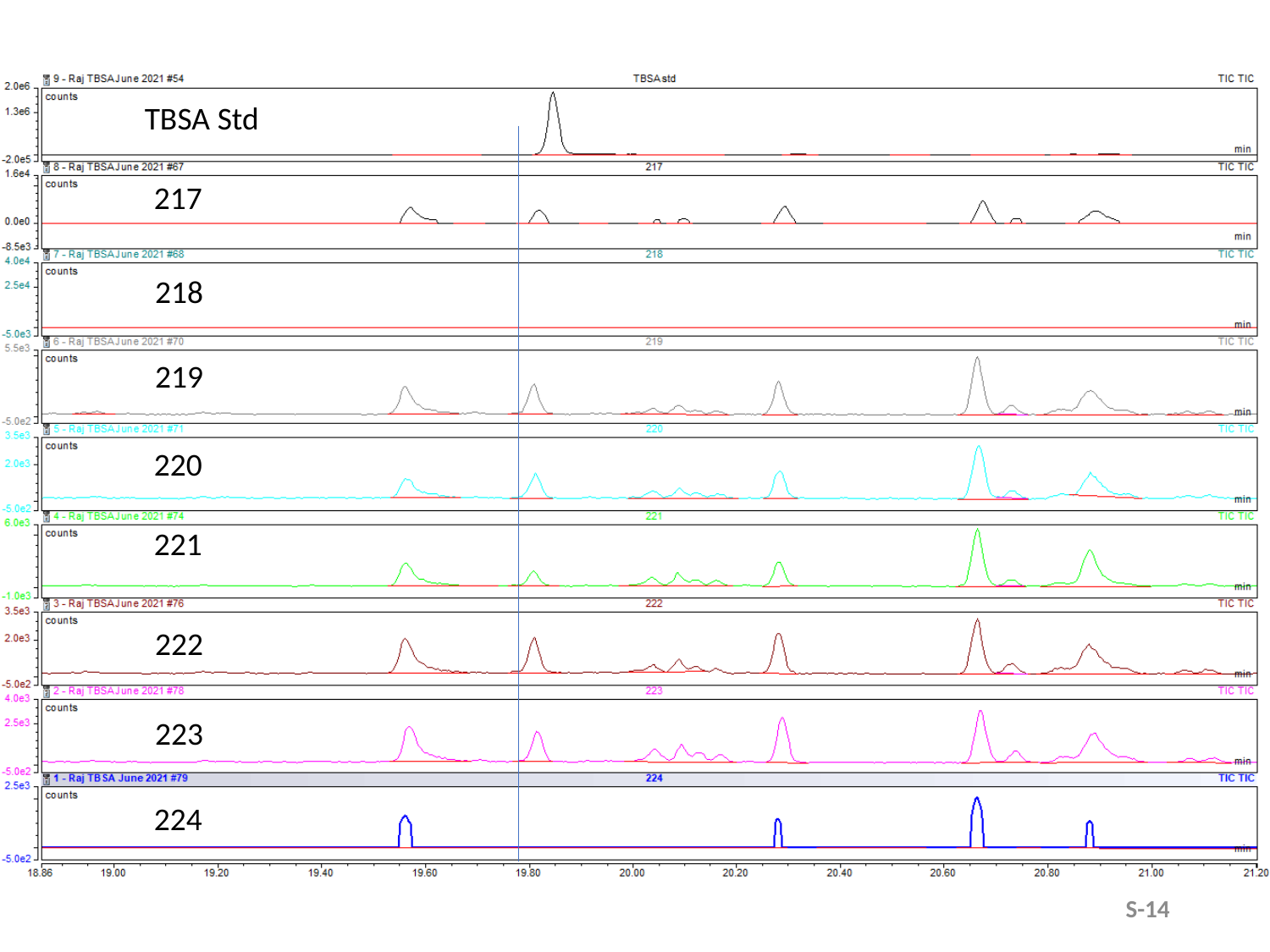

TBSA Std
217
218
219
220
221
222
223
224
S-14

## Slide 13
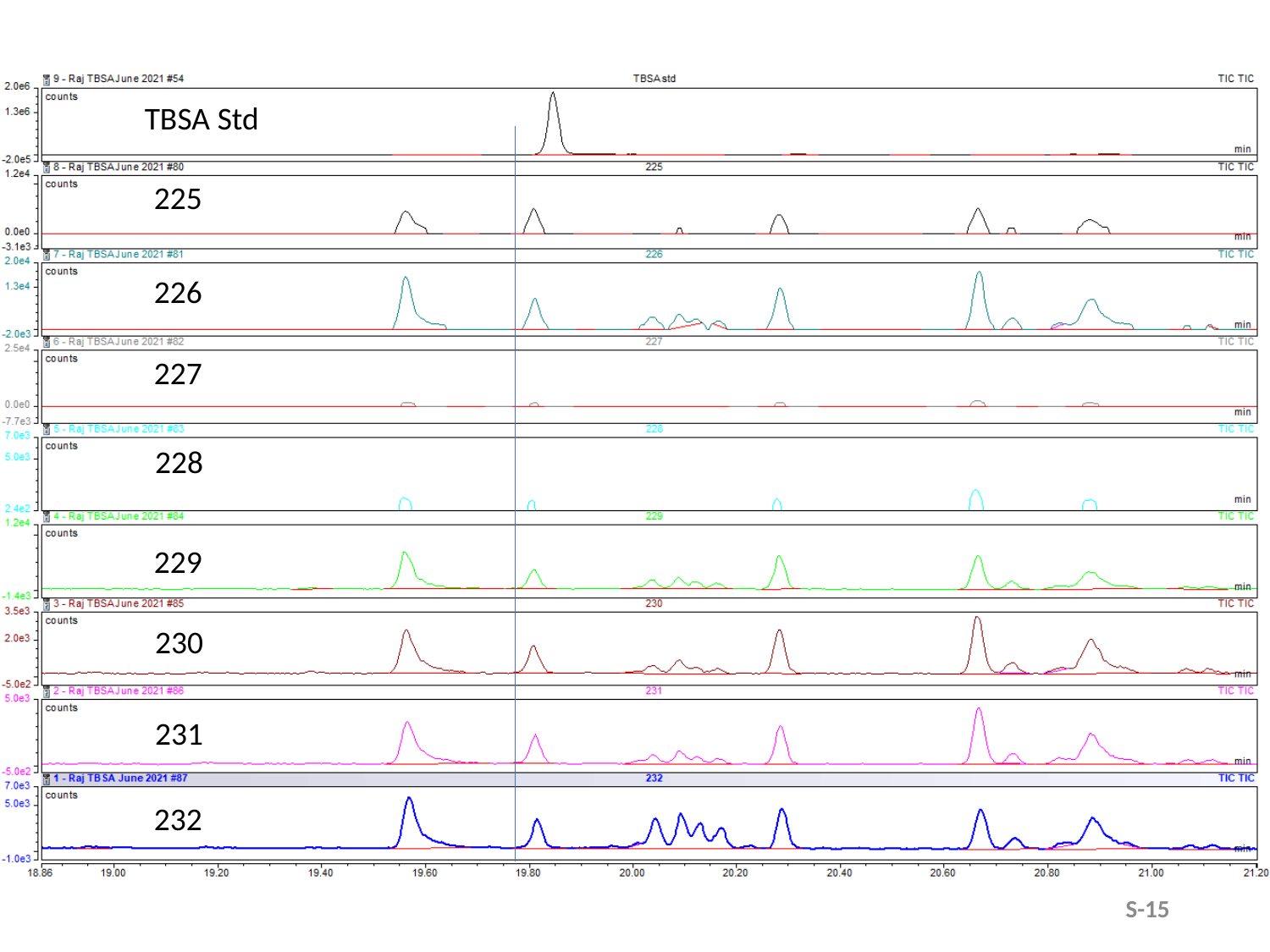

TBSA Std
225
226
227
228
229
230
231
232
S-15

## Slide 14
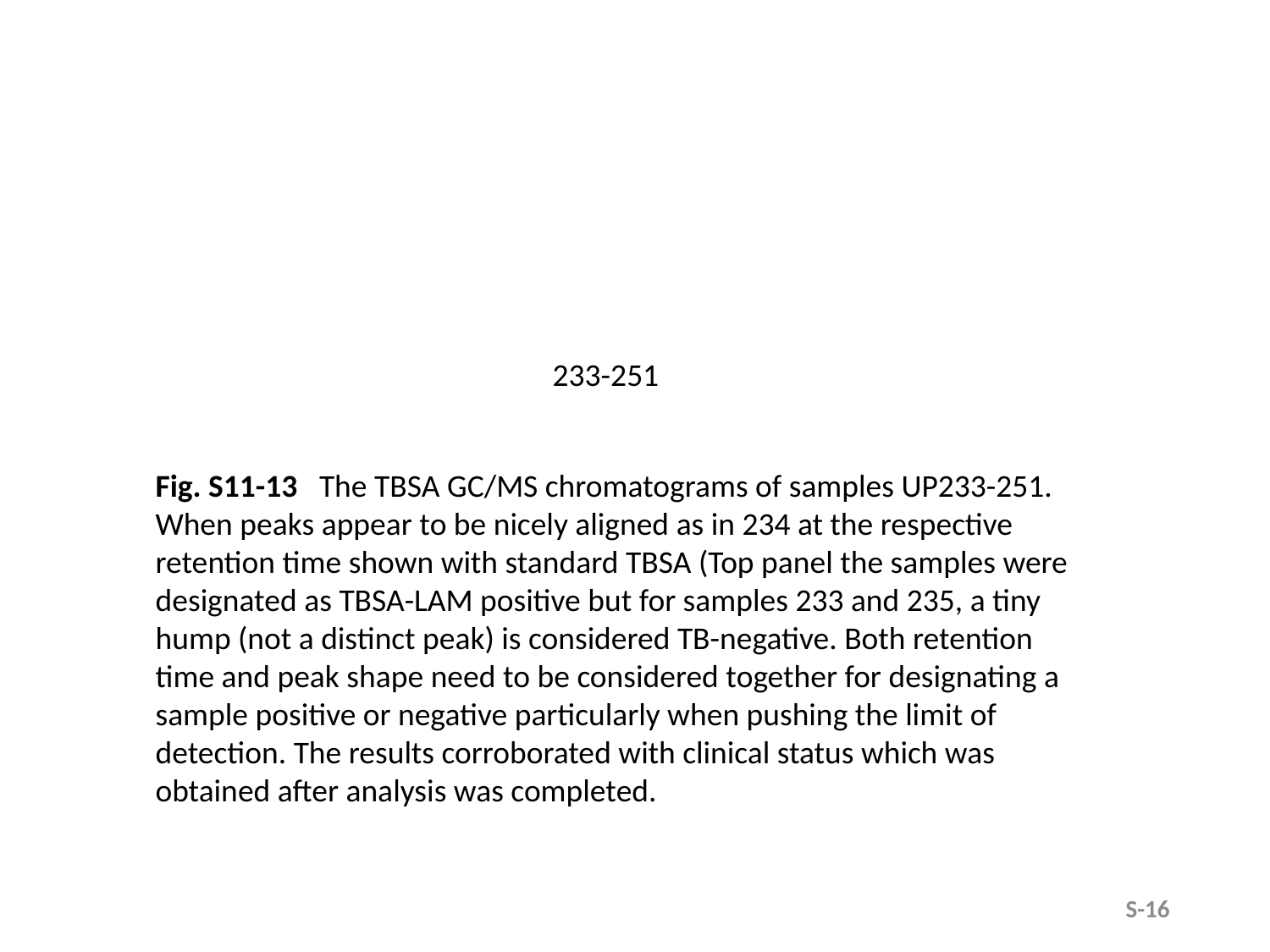

233-251
Fig. S11-13  The TBSA GC/MS chromatograms of samples UP233-251. When peaks appear to be nicely aligned as in 234 at the respective retention time shown with standard TBSA (Top panel the samples were designated as TBSA-LAM positive but for samples 233 and 235, a tiny hump (not a distinct peak) is considered TB-negative. Both retention time and peak shape need to be considered together for designating a sample positive or negative particularly when pushing the limit of detection. The results corroborated with clinical status which was obtained after analysis was completed.
S-16

## Slide 15
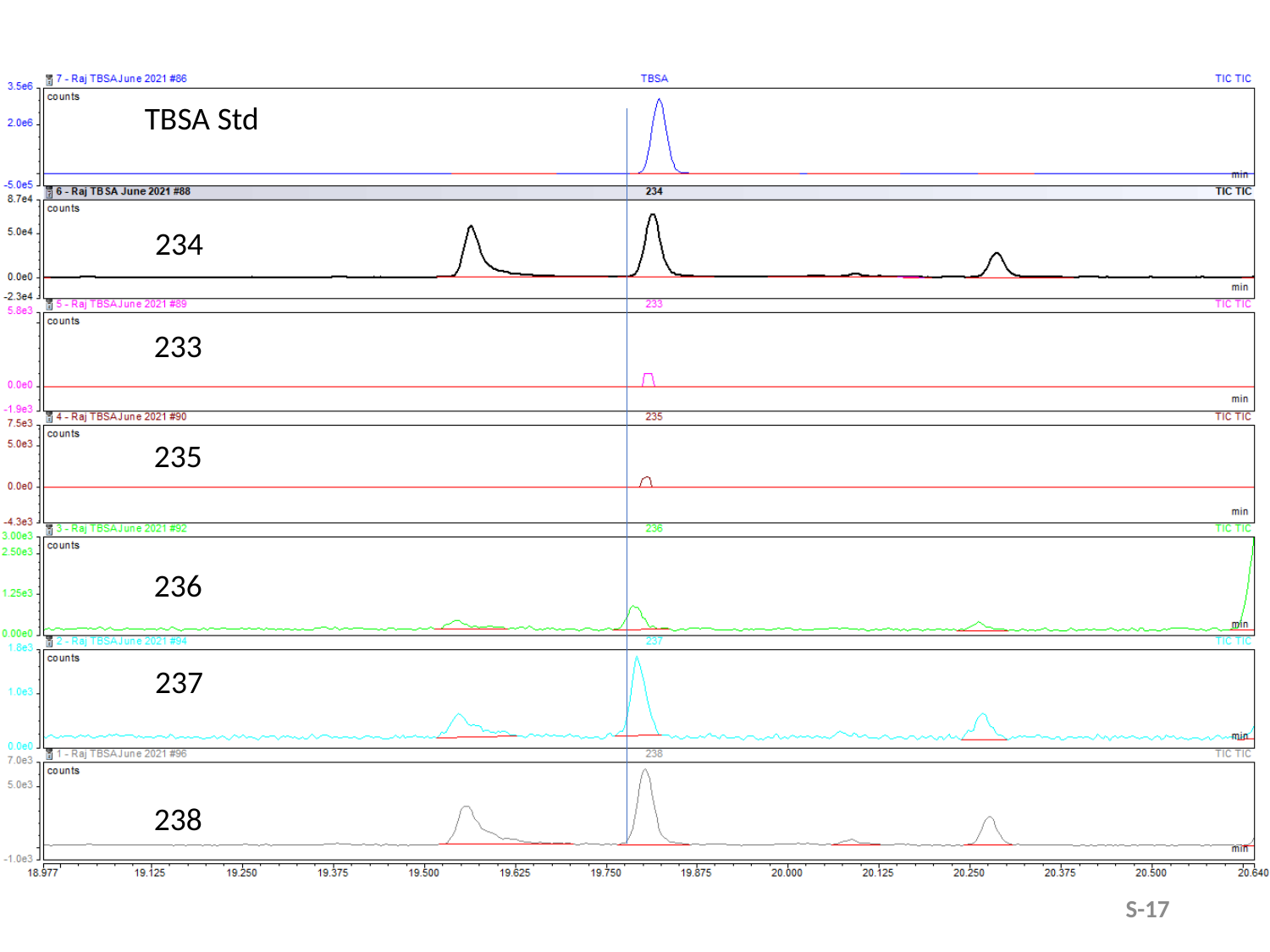

TBSA Std
234
233
235
236
237
238
S-17

## Slide 16
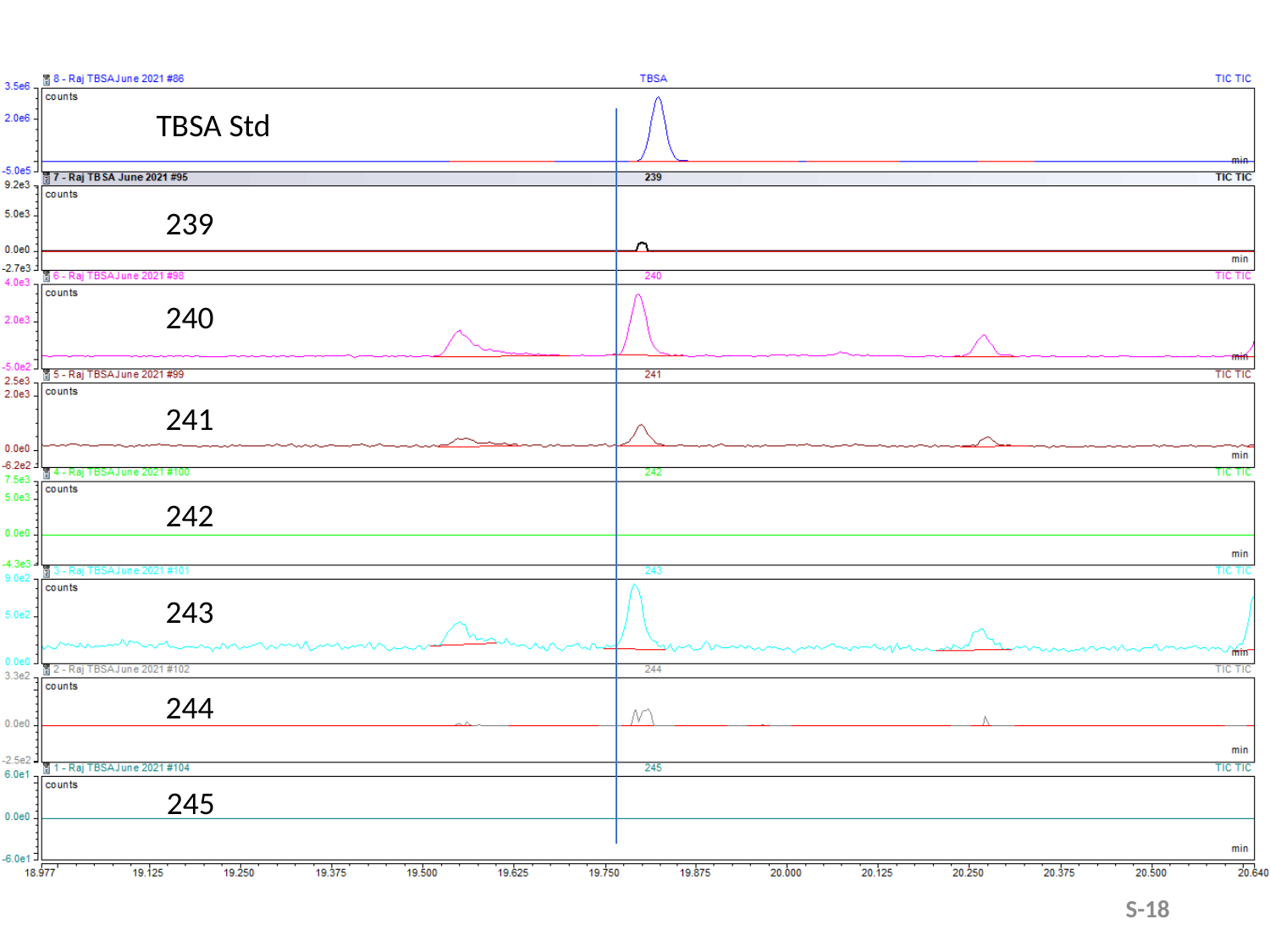

TBSA Std
239
240
241
242
243
244
245
S-18

## Slide 17
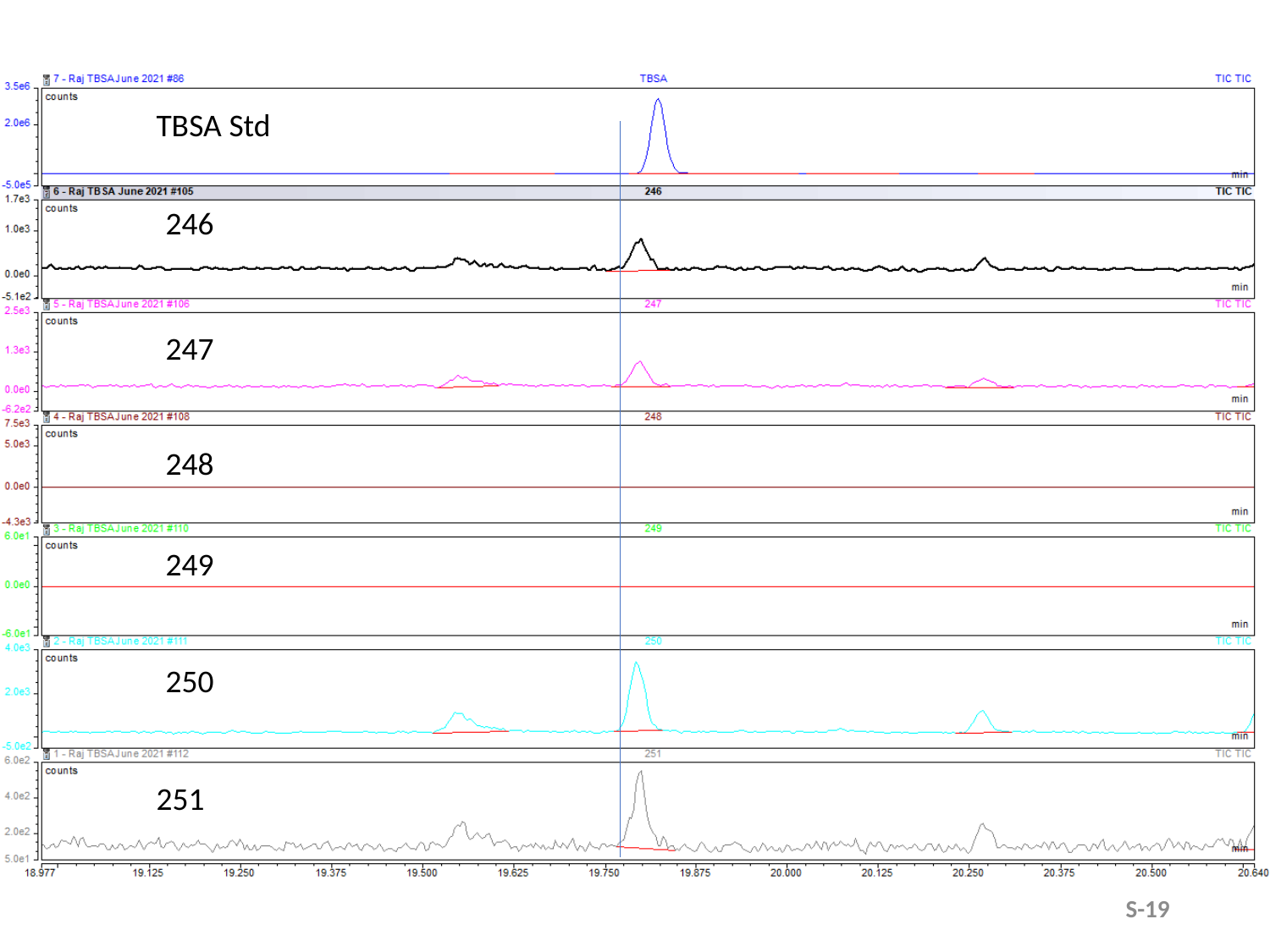

TBSA Std
246
247
248
249
250
251
S-19
