# Supplementary figures and images for: Overcome low levels of detection limit and choice of antibody affects detection of lipoarabinomannan in pediatric tuberculosis
Source: PLoS One. 2022 Oct 11;17(10):e0275838. doi: 10.1371/journal.pone.0275838 (PMC9553055; doi:10.1371/journal.pone.0275838)

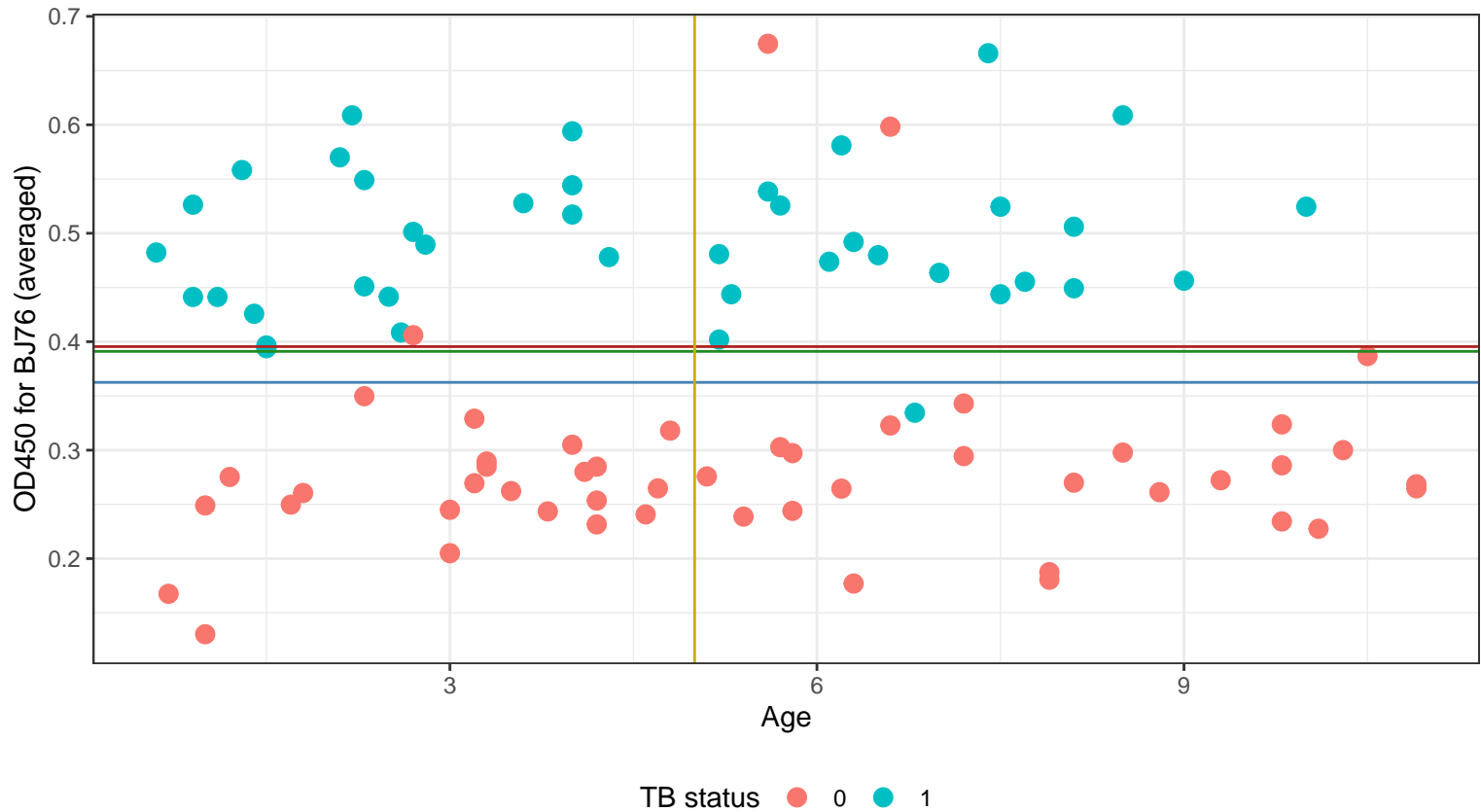

Supplement: S2 File — (PDF) [file pone.0275838.s003.pdf]
