# Supplementary material for: Overcome low levels of detection limit and choice of antibody affects detection of lipoarabinomannan in pediatric tuberculosis
Source: PLoS One. 2022 Oct 11;17(10):e0275838. doi: 10.1371/journal.pone.0275838 (PMC9553055; doi:10.1371/journal.pone.0275838)
Supplement: Attachment — Submitted filename: Response to Reviewers-2Mod.docx [file pone.0275838.s006.docx]

To The Editor

PLOS One

Ref Manuscript [PONE-D-22-15293]

Thank you for a very thorough review of our manuscript in PLOS One. **[PONE-D-22-15293]**

We have now carefully edited, reduced the Introduction by 10% and answered to the reviewers concerns as best as possible. The responses are all hilited in red.

Please include the following items when submitting your revised manuscript:

- A rebuttal letter that responds to each point raised by the academic editor and reviewer(s). You should upload this letter as a separate file labeled 'Response to Reviewers'.
- A marked-up copy of your manuscript that highlights changes made to the original version. You should upload this as a separate file labeled 'Revised Manuscript with Track Changes'.
- An unmarked version of your revised paper without tracked changes. You should upload this as a separate file labeled 'Manuscript'..

  We look forward to receiving your revised manuscript.

Journal Requirements:

When submitting your revision, we need you to address these additional requirements.

1. Please ensure that your manuscript meets PLOS ONE's style requirements, including those for file naming. The PLOS ONE style templates can be found at

[https://journals.plos.org/plosone/s/file?id=wjVg/PLOSOne_formatting_sample_main_body.pdf](https://nam10.safelinks.protection.outlook.com/?url=https%3A%2F%2Fjournals.plos.org%2Fplosone%2Fs%2Ffile%3Fid%3DwjVg%2FPLOSOne_formatting_sample_main_body.pdf&data=05%7C01%7Cdelphi.chatterjee%40colostate.edu%7C5c4796d619c043ced29d08da8c2e077f%7Cafb58802ff7a4bb1ab21367ff2ecfc8b%7C0%7C0%7C637976427336684578%7CUnknown%7CTWFpbGZsb3d8eyJWIjoiMC4wLjAwMDAiLCJQIjoiV2luMzIiLCJBTiI6Ik1haWwiLCJXVCI6Mn0%3D%7C3000%7C%7C%7C&sdata=hRGfhiakKlONMVI5aIxd0NqAHjJjx9E6CZhKhEfacQE%3D&reserved=0)  and

[https://journals.plos.org/plosone/s/file?id=ba62/PLOSOne_formatting_sample_title_authors_affiliations.pdf](https://nam10.safelinks.protection.outlook.com/?url=https%3A%2F%2Fjournals.plos.org%2Fplosone%2Fs%2Ffile%3Fid%3Dba62%2FPLOSOne_formatting_sample_title_authors_affiliations.pdf&data=05%7C01%7Cdelphi.chatterjee%40colostate.edu%7C5c4796d619c043ced29d08da8c2e077f%7Cafb58802ff7a4bb1ab21367ff2ecfc8b%7C0%7C0%7C637976427336840808%7CUnknown%7CTWFpbGZsb3d8eyJWIjoiMC4wLjAwMDAiLCJQIjoiV2luMzIiLCJBTiI6Ik1haWwiLCJXVCI6Mn0%3D%7C3000%7C%7C%7C&sdata=DJweMb88RnSxzdS7wkDRDwrEj61kUtQt9lG4ES0Rlog%3D&reserved=0)

2. Thank you for stating the following in the Acknowledgments Section of your manuscript:

“This work was funded through NIH AI R01 AI132680 (to DC) and the samples analyzed in this study were provided under NIH/NAID U19 AI109755 to Dr. Molly F Franke (Harvard Medical School).”

This statement has been removed from the acknowledgement section

We note that you have provided additional information within the Acknowledgements Section that is not currently declared in your Funding Statement. Please note that funding information should not appear in the Acknowledgments section or other areas of your manuscript. We will only publish funding information present in the Funding Statement section of the online submission form.

Please remove any funding-related text from the manuscript and let us know how you would like to update your Funding Statement. Currently, your Funding Statement reads as follows:

 “NO The funders had no role in study design, data collection and analysis, decision to publish, or preparation of the manuscript.”

This statement in correct as stated, funding related statement has been removed from the Acknowledgement section of the Manuscript

Please include your amended statements within your cover letter; we will change the online submission form on your behalf.

3. We note that the grant information you provided in the ‘Funding Information’ and ‘Financial Disclosure’ sections do not match.

When you resubmit, please ensure that you provide the correct grant numbers for the awards you received for your study in the ‘Funding Information’ section.

Funding Information provided is correct.

4. We note that you have stated that you will provide repository information for your data at acceptance. Should your manuscript be accepted for publication, we will hold it until you provide the relevant accession numbers or DOIs necessary to access your data. If you wish to make changes to your Data Availability statement, please describe these changes in your cover letter and we will update your Data Availability statement to reflect the information you provide.

The Supplementary Files (Supporting Information) provided with this manuscript contain all the data presented in this study. There is no other repository information for data availability.

5. Please include captions for your Supporting Information files at the end of your manuscript, and update any in-text citations to match accordingly. Please see our Supporting Information guidelines for more information: [http://journals.plos.org/plosone/s/supporting-information](https://nam10.safelinks.protection.outlook.com/?url=http%3A%2F%2Fjournals.plos.org%2Fplosone%2Fs%2Fsupporting-information&data=05%7C01%7Cdelphi.chatterjee%40colostate.edu%7C5c4796d619c043ced29d08da8c2e077f%7Cafb58802ff7a4bb1ab21367ff2ecfc8b%7C0%7C0%7C637976427336840808%7CUnknown%7CTWFpbGZsb3d8eyJWIjoiMC4wLjAwMDAiLCJQIjoiV2luMzIiLCJBTiI6Ik1haWwiLCJXVCI6Mn0%3D%7C3000%7C%7C%7C&sdata=9Cj3kpJU07cCoPCcKXs3z2woRPcAqXUJEsfOHtBBTJQ%3D&reserved=0).

Captions for supporting Information are revised and included at the end of the manuscript after the References and updated in-text citations to match

6. Please review your reference list to ensure that it is complete and correct. If you have cited papers that have been retracted, please include the rationale for doing so in the manuscript text, or remove these references and replace them with relevant current references. Any changes to the reference list should be mentioned in the rebuttal letter that accompanies your revised manuscript. If you need to cite a retracted article, indicate the article’s retracted status in the References list and also include a citation and full reference for the retraction notice.
Not Applicable, All References have been cross checked.

[Note: HTML markup is below. Please do not edit.]

Reviewers' comments:

Reviewer's Responses to Questions

**Comments to the Author**

1. Is the manuscript technically sound, and do the data support the conclusions?

The manuscript must describe a technically sound piece of scientific research with data that supports the conclusions. Experiments must have been conducted rigorously, with appropriate controls, replication, and sample sizes. The conclusions must be drawn appropriately based on the data presented.

Reviewer #1: Yes

Reviewer #2: Yes

2. Has the statistical analysis been performed appropriately and rigorously?

Reviewer #1: Yes

Reviewer #2: Yes

3. Have the authors made all data underlying the findings in their manuscript fully available?

The [PLOS Data policy](https://nam10.safelinks.protection.outlook.com/?url=http%3A%2F%2Fwww.plosone.org%2Fstatic%2Fpolicies.action%23sharing&data=05%7C01%7Cdelphi.chatterjee%40colostate.edu%7C5c4796d619c043ced29d08da8c2e077f%7Cafb58802ff7a4bb1ab21367ff2ecfc8b%7C0%7C0%7C637976427336840808%7CUnknown%7CTWFpbGZsb3d8eyJWIjoiMC4wLjAwMDAiLCJQIjoiV2luMzIiLCJBTiI6Ik1haWwiLCJXVCI6Mn0%3D%7C3000%7C%7C%7C&sdata=%2BSIvcnHn9vrvrVBOQDGbtBazHEpnHWHAOD5%2FrqUJa30%3D&reserved=0) requires authors to make all data underlying the findings described in their manuscript fully available without restriction, with rare exception (please refer to the Data Availability Statement in the manuscript PDF file). The data should be provided as part of the manuscript or its supporting information, or deposited to a public repository. For example, in addition to summary statistics, the data points behind means, medians and variance measures should be available. If there are restrictions on publicly sharing data—e.g. participant privacy or use of data from a third party—those must be specified.

Reviewer #1: Yes

Reviewer #2: Yes

4. Is the manuscript presented in an intelligible fashion and written in standard English?

PLOS ONE does not copyedit accepted manuscripts, so the language in submitted articles must be clear, correct, and unambiguous. Any typographical or grammatical errors should be corrected at revision, so please note any specific errors here.

Reviewer #1: Yes

Reviewer #2: Yes

5. Review Comments to the Author

Please use the space provided to explain your answers to the questions above. You may also include additional comments for the author, including concerns about dual publication, research ethics, or publication ethics. (Please upload your review as an attachment if it exceeds 20,000 characters)

Reviewer #1: i review with interest the study titled "Overcome Low Levels of Detection Limit and Choice of Antibody Affects Detection of
Lipoarabinomannan in Pediatric Tuberculosis" on 91 urine samples from children aged 3-10 years. urine samples were analyzed for tuberculostearic acid (TBSA) by gas chromatography/mass spectrometry (GC/MS) and
capture ELISA (C-ELISA). the study is interesting and well written. but i have few comments that could improve the manuscript:
1- the introduction is too long and should be shortened. Edited and shortened
2- write the type of the study? Experimental Assay Assessment
3- what is the duration of the study? Samples were from the U19 (NIH/NAID U19 AI109755) program that originated in 2015,-Mesman, A.W., Soto, M., Coit, J. *et al.* Detection of *Mycobacterium tuberculosis* in pediatric stool samples using TruTip technology. *BMC Infect Dis* **19**, 563 (2019). <https://doi.org/10.1186/s12879-019-4188-8>. Children recruited to participate in a pediatric TB Diagnostic Study between May 2015-Feb 2018 in Peru.

91 urine sample cohort was sent to CSU in 2020, GC/MS was done in September 2021and C-ELISA completed in March 2022, manuscript incorporating all data was submitted in May 2022. Thus, the duration of experimental work described herein was 2020-2022.
4- what are the exclusion criteria? For our study-Not Applicable as the study design was applied by the U19 program which states. “Subjects recruited in the pediatric TB diagnostics study were less than 15 years of age with a history of contact with an adult with TB within the previous two years, presenting with TB like symptoms i.e., persistent cough for two weeks or longer, unexplained weight loss and fever with fatigue for more than a week.
5- what are the primary and secondary outcomes of the study? The primary outcome of our study is LAM is present albeit in low levels in children with TB symptoms as tested by clinicians irrespective of culture/smear status and amounts of LAM is quantifiable by GC/MS. Secondary outcome is, sensitive immunoassay needs to be developed with more selective Abs, or sample concentration need to be performed to detect low levels of urinary LAM among children
6- how did you estimate the sample size? and what is the power of the study? Sample size was not predetermined, we received and analyzed what was available as left over and sent to CSU for our experiments.

Reviewer #2: Challenges with specimen collection and bacteriological confirmation of TB in young children, due to the paucibacillary nature of TB disease in this age group and the lack of highly sensitive point-of care tests. There is no gold-standard test available for children, and many of the older testing methods are often time-consuming and inaccurate. Using urine samples is a non-invasive, non-sputum-based diagnostic assay and useful method of detecting TB in children. The study indicates that for pediatric TB diagnosis, an ELISA based assay (which uses larger sample volumes) may be more appropriate where sample concentration can be applied. All children samples in this study were smear negative make the positive results in urine sample is more valuable. The study populations were very clear and appropriately. TB group, and non TB group based on 5 combination parameters: clinical symptom, X-ray, exposure history, TST and bacteriological evidence (microscopy, culture). Properly designed positive and negative control samples. Urine positive control for LAM spiked with CDC1551 LAM. The same urine sample was used as a negative control (no spike) for the urine background to derive a cutoff for the analysis of clinical samples. The used antibodies (mouse mAb CS35 IgG3, mAb A194-01 IgG1) were well calibrated. Techniques (C-ELISA , GC/MS) applied according to standard procedures. The statistical analysis has been performed appropriately and rigorously (BJ76 was tested twice and the OD450 values averaged. Confidence intervals for sensitivity and specificity were calculated using Wilson’s method. Statistical analyses were conducted in R open source software version 4.1.1). In 2022, WHO strongly recommends using LF-LAM to assist in the diagnosis of TB disease in HIV-positive adults and children with signs and symptoms of TB (pulmonary and/or extrapulmonary). The result of study make a suggestion that the use of urine specimens may consider as a tool for definitive diagnosis of tuberculosis, not only as a diagnostic assist. Unfortunately, there was no HIV status of the pediatric patient in the study. If the author analyzes the results by age group, especially in the group of children under 5 years old, it may have an additional contribution in screening, diagnosis and treatment. We agree with the reviewer, the shortfall of our study is that we had no children sample with HIV coinfection for comparison. When we initiated this work, our goal was to thoroughly investigate LAM distribution among HIV negative TB positive patients and we concentrated in samples originating in Peru where HIV incidence is >1%. The children cohort also originated in Peru, therefore, no HIV, we are now looking into a new pediatric sample source which will have HIV/TB coinfection. As suggested, we analyzed the data based on <5yrs>, and our analysis shows that LAM amounts are equally distributed among all ages. (see **forest plot in Supplementary Fig S14**). This also, fits our hypothesis that when low levels (+/-2-4ng) of LAM are detected, it is difficult to correlate with any factors such as age/smear and perhaps sex.

6. PLOS authors have the option to publish the peer review history of their article ([what does this mean?](https://nam10.safelinks.protection.outlook.com/?url=https%3A%2F%2Fjournals.plos.org%2Fplosone%2Fs%2Feditorial-and-peer-review-process%23loc-peer-review-history&data=05%7C01%7Cdelphi.chatterjee%40colostate.edu%7C5c4796d619c043ced29d08da8c2e077f%7Cafb58802ff7a4bb1ab21367ff2ecfc8b%7C0%7C0%7C637976427336840808%7CUnknown%7CTWFpbGZsb3d8eyJWIjoiMC4wLjAwMDAiLCJQIjoiV2luMzIiLCJBTiI6Ik1haWwiLCJXVCI6Mn0%3D%7C3000%7C%7C%7C&sdata=HE9DIniVPTWVzznP61ElEt1%2B1Dhb%2BitCUOkxGv0gGOM%3D&reserved=0)). If published, this will include your full peer review and any attached files.


If you choose “no”, your identity will remain anonymous but your review may still be made public.


**Do you want your identity to be public for this peer review?** For information about this choice, including consent withdrawal, please see our [Privacy Policy](https://nam10.safelinks.protection.outlook.com/?url=https%3A%2F%2Fwww.plos.org%2Fprivacy-policy&data=05%7C01%7Cdelphi.chatterjee%40colostate.edu%7C5c4796d619c043ced29d08da8c2e077f%7Cafb58802ff7a4bb1ab21367ff2ecfc8b%7C0%7C0%7C637976427336840808%7CUnknown%7CTWFpbGZsb3d8eyJWIjoiMC4wLjAwMDAiLCJQIjoiV2luMzIiLCJBTiI6Ik1haWwiLCJXVCI6Mn0%3D%7C3000%7C%7C%7C&sdata=EpUZ3KbrYKef3ulRPG%2F1WMFbMfqtkvfCI9IOJE8i%2Fwk%3D&reserved=0).

Reviewer #1: **Yes:** Doaa El Amrousy

Reviewer #2: No

[NOTE: If reviewer comments were submitted as an attachment file, they will be attached to this email and accessible via the submission site. Please log into your account, locate the manuscript record, and check for the action link "View Attachments". If this link does not appear, there are no attachment files.]

While revising your submission, please upload your figure files to the Preflight Analysis and Conversion Engine (PACE) digital diagnostic tool, [https://pacev2.apexcovantage.com/](https://nam10.safelinks.protection.outlook.com/?url=https%3A%2F%2Fpacev2.apexcovantage.com%2F&data=05%7C01%7Cdelphi.chatterjee%40colostate.edu%7C5c4796d619c043ced29d08da8c2e077f%7Cafb58802ff7a4bb1ab21367ff2ecfc8b%7C0%7C0%7C637976427336840808%7CUnknown%7CTWFpbGZsb3d8eyJWIjoiMC4wLjAwMDAiLCJQIjoiV2luMzIiLCJBTiI6Ik1haWwiLCJXVCI6Mn0%3D%7C3000%7C%7C%7C&sdata=fgXChJh7YT9nl7XKZ1P1GS133s9gqInwxVelmbBXWZQ%3D&reserved=0). PACE helps ensure that figures meet PLOS requirements. To use PACE, you must first register as a user. Registration is free. Then, login and navigate to the UPLOAD tab, where you will find detailed instructions on how to use the tool. If you encounter any issues or have any questions when using PACE, please email PLOS at [figures@plos.org](mailto:figures@plos.org). Please note that Supporting Information files do not need this step.

*In compliance with data protection regulations, you may request that we remove your personal registration details at any time.* [*(Remove my information/details)*](https://nam10.safelinks.protection.outlook.com/?url=https%3A%2F%2Fwww.editorialmanager.com%2Fpone%2Flogin.asp%3Fa%3Dr&data=05%7C01%7Cdelphi.chatterjee%40colostate.edu%7C5c4796d619c043ced29d08da8c2e077f%7Cafb58802ff7a4bb1ab21367ff2ecfc8b%7C0%7C0%7C637976427336840808%7CUnknown%7CTWFpbGZsb3d8eyJWIjoiMC4wLjAwMDAiLCJQIjoiV2luMzIiLCJBTiI6Ik1haWwiLCJXVCI6Mn0%3D%7C3000%7C%7C%7C&sdata=VJLVDWvsQQ%2BLuPlYSs8ERRK9ax9GaC4ged5teeEr7dk%3D&reserved=0)*. Please contact the publication office if you have any questions.*
